# C/EBPδ as a Regulatory Node in Adipocytes: Roles in Differentiation, Metabolism, and Immune Function

**DOI:** 10.3390/biom16050641

**Published:** 2026-04-24

**Authors:** Suining Ma, Meiting Lai, Tongjun Li, Lexun Wang, Xianglu Rong

**Affiliations:** Key Laboratory of Glucolipid Metabolic Diseases, Ministry of Education; Guangdong Provincial TCM Key Laboratory for Metabolic Diseases; Guangdong Provincial Research Center of Integration of Traditional Chinese Medicine and Western Medicine in Metabolic Diseases; Guangdong Pharmaceutical University, Guangzhou 510006, China

**Keywords:** C/EBPδ, adipocyte differentiation, adipogenesis, immune regulation, context dependence

## Abstract

CCAAT/enhancer-binding protein δ (C/EBPδ) is a rapidly responsive transcription factor that occupies an important regulatory position in adipocytes. Induced during the early stage of adipocyte differentiation, C/EBPδ integrates hormonal, inflammatory, metabolic, and stress-related cues and contributes to the coordination of downstream transcriptional and functional programs. Beyond its role in the initiation of differentiation, C/EBPδ is also involved in adipogenic progression, metabolic regulation, and immune-related functions in adipocytes. Current evidence indicates that C/EBPδ participates in early adipogenic regulatory networks, contributes to lipid metabolic programs, and is associated with immune-regulatory processes linked to lipid antigen presentation. Notably, the biological output of C/EBPδ is strongly shaped by tissue type, developmental stage, and microenvironmental context, ranging from promotion of adipogenic differentiation to regulation of inflammatory, metabolic, and adaptive stress responses under distinct physiological and pathological conditions. This review summarizes the upstream regulatory network, downstream functional framework, and context-dependent roles of C/EBPδ in adipocytes, and further discusses its potential relevance to adipose-related diseases as well as the opportunities and challenges for future precision intervention strategies.

## 1. Introduction

Adipose tissue has long been regarded primarily as an energy storage depot; however, accumulating evidence has greatly expanded our understanding of its biological functions. It is now widely recognized as a highly active metabolic regulatory hub and endocrine organ that plays indispensable roles in systemic energy homeostasis, insulin sensitivity, inflammatory regulation, and immune responses [1]. Mature adipocytes not only secrete a variety of adipokines, such as leptin and adiponectin, but also engage in complex interactions with tissue-resident and infiltrating immune cells, thereby coordinately shaping local and systemic metaflammation [1]. Adipose tissue dysfunction, characterized by defective adipocyte differentiation and altered adipokine secretion, is widely recognized as a critical factor in the development and progression of metabolic disorders such as obesity, insulin resistance, type 2 diabetes mellitus, and non-alcoholic fatty liver disease [2,3]. These features suggest that transcriptional regulators capable of integrating differentiation, metabolic, and immune-related signals may play particularly important roles in adipocyte biology.

Adipogenesis is a complex, multi-stage biological process governed by a cascade of transcription factors, encompassing the lineage progression from mesenchymal stem cells to committed preadipocytes and ultimately to fully differentiated, functional adipocytes [4]. In contrast, adipocyte differentiation typically refers specifically to the terminal stage in which preadipocytes mature into adipocytes and is therefore considered the core step of adipogenesis [4,5,6]. During this process, a tightly coordinated transcriptional network orchestrates adipocyte development. Among these regulators, peroxisome proliferator-activated receptor gamma (PPARγ) is widely recognized as the master regulator of adipogenesis, while members of the CCAAT/enhancer-binding protein (C/EBP) family cooperate with PPARγ to drive and maintain the expression of adipocyte-specific genes [7,8]. In the early phase of differentiation, C/EBPβ (encoded by *CEBPB*) initiates the transcription of PPARγ and C/EBPα (encoded by *CEBPA*). The positive feedback loop formed between PPARγ and C/EBPα subsequently stabilizes the adipogenic transcriptional program and is essential for maintaining the phenotype of mature adipocytes [9].

During the initiation of adipocyte differentiation, C/EBPδ (encoded by *CEBPD*), another member of the C/EBP family, is rapidly upregulated in parallel with C/EBPβ and serves as an early transcriptional regulator. Under stimulation by the classical MDI induction cocktail, consisting of 3-isobutyl-1-methylxanthine (IBMX), dexamethasone (DEX), and insulin, both factors are upregulated and cooperatively initiate the transcription of downstream target genes, including the induction of PPARγ and C/EBPα expression [10]. However, the role of these early C/EBP factors is not fully explained by a simple linear transcriptional cascade. In vivo studies of C/EBPβ/C/EBPδ double-deficient mice have shown markedly impaired adipocyte differentiation and adipose tissue development, supporting the view that early C/EBP inputs are important but not adequately captured by a strictly sequential hierarchy [11]. Notably, although C/EBPβ and C/EBPδ are both rapidly induced following adipogenic stimulation, their functional activities are closely coupled to early cell-cycle remodeling during differentiation. Previous studies have shown that C/EBPδ is involved in mitotic clonal expansion (MCE) and early transcriptional regulation, suggesting that its function is tightly linked to cell-cycle progression at the initiation stage of adipocyte differentiation [12,13]. Thus, C/EBPδ is not merely an early marker of adipocyte differentiation, but also a critical regulatory node linking upstream adipogenic cues to the core downstream transcriptional cascade.

Beyond the classical transcription factor network, extracellular upstream cues and epigenetic mechanisms are also recognized as important modulators of adipogenesis and adipocyte differentiation at multiple levels. For example, the inflammatory cytokine interleukin-33 has been reported to inhibit preadipocyte differentiation by regulating Wnt/β-catenin signaling and thereby suppressing the expression of the key downstream adipogenic regulator PPARγ [14]. At the epigenetic level, alterations in the expression of protein arginine methyltransferase 6 can influence the transcriptional activity of core adipogenic regulators, including PPARγ and C/EBPα, through histone arginine methylation, thereby contributing to the epigenetic control of adipogenesis [15]. Together, these findings indicate that adipocyte differentiation is governed not only by the canonical transcriptional cascade but also by upstream signaling inputs and epigenetic regulation. Given its position at the front end of the early transcriptional response network, C/EBPδ may serve as an important integrative node for these regulatory signals.

As a member of the C/EBP family, C/EBPδ exhibits functional roles that extend well beyond its participation in the classical transcriptional network regulating adipogenesis. Increasing evidence indicates that C/EBPδ exhibits marked cell type specificity and strong dependence on the physiological or pathological microenvironment. In distinct cellular contexts and under different physiological or disease conditions, C/EBPδ can participate in a broad range of signaling responses and functional regulatory processes rather than acting within a single linear pathway [16]. This feature is particularly evident in adipocyte-related systems. Under diverse stimuli, such as hormonal signals and inflammatory mediators, as well as under different metabolic states, including energy surplus or deprivation, and in specific tissue microenvironments, such as hypoxia and aging, the expression and function of C/EBPδ show substantial heterogeneity and may even lead to opposing biological outcomes. These observations suggest that a comprehensive understanding of the role of C/EBPδ in adipocytes requires an integrated analysis within specific physiological and pathological contexts.

Although C/EBPδ has long been recognized as an early adipogenic factor, current findings regarding its role in adipocytes are still scattered across differentiation, metabolic regulation, immune-related functions, and diverse microenvironmental settings, making it difficult to capture its role within a single conventional framework. Against this background, this review aims to systematically summarize the transcriptional regulatory features and functional diversity of C/EBPδ in adipocytes. We first outline the upstream signaling networks that regulate C/EBPδ expression and transcriptional activity, and then discuss in depth its downstream biological functions and molecular mechanisms in processes including adipocyte differentiation, lipid metabolic homeostasis, and immune-related lipid antigen presentation in adipocytes. We further examine how changes in C/EBPδ expression differentially affect adipocyte function under distinct physiological states, such as differentiation stage and metabolic status, as well as under pathological conditions, including inflammation and aging. By integrating the available evidence, this review seeks to clarify the complexity and context dependence of C/EBPδ as a regulatory node in adipocyte biology, thereby providing a theoretical framework for understanding the molecular basis of adipocyte plasticity and offering potential insights into targeted intervention in obesity and related metabolic disorders.

## 2. Regulatory Network Governing C/EBPδ Expression and Activity in Adipocytes

**Figure 1:**
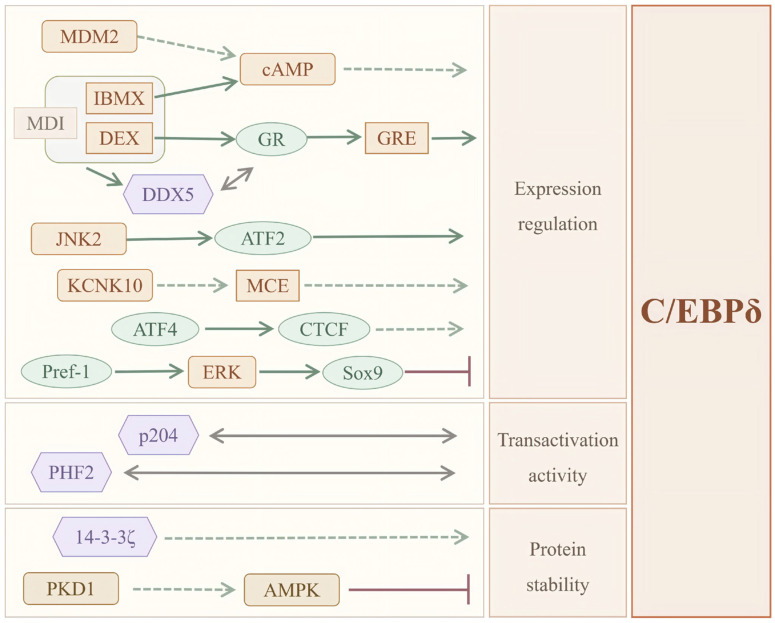
Representative upstream regulatory network controlling C/EBPδ expression and activity in adipocytes. 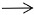 promotion; 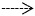 Indirect effect; 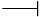 inhibition; 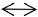 protein interaction or cooperative regulation. ATF2: Activating transcription factor 2; ATF4: Activating transcription factor 4; AMPK: AMP-activated protein kinase; C/EBPδ: CCAAT/enhancer-binding protein δ; cAMP: cyclic adenosine monophosphate; CTCF: CCCTC-binding factor; DEX: dexamethasone; DDX5: DEAD-box helicase 5; ERK: extracellular signal-regulated kinase; GR: glucocorticoid receptor; GRE: glucocorticoid response element; IBMX: 3-isobutyl-1-methylxanthine; JNK2: c-Jun N-terminal kinase 2; KCNK10: potassium two-pore domain channel subfamily K member 10; MCE: mitotic clonal expansion; MDI: differentiation-inducing cocktail containing IBMX, dexamethasone and insulin; MDM2: murine double minute 2; p204: Interferon-inducible protein 204; PHF2: plant homeodomain finger protein 2; PKD1: protein kinase D1; Pref-1: preadipocyte factor 1; Sox9: SRY-box transcription factor 9, Sox9 has also been reported to repress C/EBPβ; only the C/EBPδ branch is highlighted here for simplicity; 14-3-3ζ: 14-3-3 zeta protein. These signals regulate C/EBPδ through multiple layers, including expression regulation, transactivation activity, and protein stability, thereby influencing adipocyte differentiation.

As a key transcription factor activated in the early phase of adipocyte differentiation, C/EBPδ occupies an important position within a regulatory network linking upstream inductive signals to the downstream adipogenic transcriptional program. Its expression, protein homeostasis, and capacity to activate the transcription of downstream target genes are all subject to regulation by a multilayered network. These regulatory inputs arise from multiple levels, including differentiation-triggering signals, protein homeostasis-related regulation, cooperating transcription factors, and endogenous inhibitory mechanisms, thereby helping to ensure that C/EBPδ is induced at the appropriate time and place to exert its corresponding functions. To more clearly illustrate the major upstream regulatory cues governing C/EBPδ during adipocyte differentiation, as well as the different levels at which they act, the relevant regulatory network is summarized in Figure 1. The following sections systematically discuss these regulatory sources and outline the key upstream pathways and modes of regulation involved.

### 2.1. Multiple Activating Signals at the Onset of Adipocyte Differentiation

At the initiation of adipocyte differentiation, multiple extracellular and intracellular activating signals contribute to the rapid expression of C/EBPδ, thereby establishing the molecular basis for subsequent activation of the C/EBP–PPARγ core transcriptional cascade. In particular, C/EBPδ is rapidly upregulated within hours after stimulation with the classical MDI induction cocktail. Available evidence indicates that this induction process is closely associated with dexamethasone (DEX)-responsive signaling mediated by the glucocorticoid receptor (GR), while also being influenced by 3-isobutyl-1-methylxanthine (IBMX)/cyclic adenosine monophosphate (cAMP)-related pathways [17,18]. In addition, mouse double minute 2 (MDM2) has been implicated in supporting the early induction of C/EBPδ during the initial phase of differentiation. Its effect appears to be associated with cAMP-related Janus kinase/signal transducer and activator of transcription 3 signaling and CREB-regulated transcription coactivator (CRTC)-mediated transcriptional activation, suggesting that MDM2 plays a supportive role in promoting C/EBPδ transcription during the early differentiation window [19,20].

GR represents one of the best-characterized direct upstream regulators of C/EBPδ expression during the early phase of differentiation. Following activation by dexamethasone (DEX), GR translocates to the nucleus and directly interacts with glucocorticoid response elements (GREs) in the C/EBPδ promoter, thereby promoting its transcription [17,21]. DEAD-box RNA helicase 5 (DDX5) can interact with GR under MDI induction conditions and facilitate the GR-mediated early induction of C/EBPδ, thereby contributing to the upstream activating signals of C/EBPδ during the initiation phase of differentiation [17,22]. In addition, the c-Jun N-terminal kinase (JNK) pathway represents another important upstream route implicated in C/EBPδ transactivation during early differentiation. In particular, JNK2 activates activating transcription factor 2 (ATF2) through phosphorylation, which enhances ATF2 binding to the C/EBPδ promoter and increases its transactivation activity, ultimately promoting the early induction of C/EBPδ [23].

Beyond these pathways, activating transcription factor 4 (ATF4) also contributes to transcriptional regulation during the early stage of adipocyte differentiation. ATF4 has been shown to directly regulate the expression of CCCTC-binding factor (CTCF), and ATF4 co-localizes with CTCF at the promoter region of the *CEBPD* gene, where the two factors cooperatively enhance promoter activity and thereby promote C/EBPδ expression [24]. Meanwhile, potassium two-pore domain channel subfamily K member 10 (KCNK10) has been implicated in mitotic clonal expansion during early differentiation and has been associated with altered expression of both C/EBPβ and C/EBPδ, thereby indirectly contributing to the induction of C/EBPδ at this stage [12]. Collectively, these positive regulatory mechanisms help support the rapid and efficient induction of C/EBPδ during the early phase of adipogenic stimulation, thereby enabling the orderly initiation of the core transcriptional program that drives adipocyte differentiation.

### 2.2. Regulation of C/EBPδ Function by Protein Homeostasis

During adipocyte differentiation, in addition to transcriptional induction, the maintenance of C/EBPδ protein homeostasis is another important determinant of its functional activity. The adaptor protein 14-3-3ζ (14-3-3 zeta) has been shown to stabilize C/EBPδ by modulating autophagy–lysosome–associated processes. In contrast, loss of 14-3-3ζ reduces C/EBPδ protein abundance and impairs its nuclear localization, thereby impairing the early program of adipocyte differentiation [25]. In addition, protein kinase D1 (PKD1), a negative regulator of adipocyte energy metabolism and beige adipocyte formation, has been reported to reduce C/EBPδ protein levels through an AMP-activated protein kinase (AMPK)-dependent pathway. This reduction is associated with decreased expression of β3-adrenergic receptor and several genes associated with beige adipocyte differentiation, thereby favoring a phenotype more inclined toward energy storage [26]. Overall, the abundance of C/EBPδ protein appears to be maintained through a dynamic balance between stabilization and degradation. This balance, together with early transcriptional induction, contributes to the functional activity of C/EBPδ during adipocyte differentiation and related metabolic adaptations.

### 2.3. Enhancement of C/EBPδ Function by Cooperating Factors

In addition to transcriptional induction and protein stability control, the effective activity of C/EBPδ can also be enhanced by several cooperating factors. Interferon-inducible protein 204 is induced during the initial phase of adipocyte differentiation and can directly interact with C/EBPδ, thereby enhancing its transcriptional activation of downstream adipogenic genes. Interferon-inducible protein 204 therefore acts as an important cooperative regulator during the early phase of adipocyte differentiation [27]. Meanwhile, plant homeodomain finger protein 2 (PHF2), a histone demethylase that specifically removes histone H3 lysine 9 dimethylation, can interact with C/EBPδ and promote chromatin accessibility at adipogenic gene promoters through H3 lysine 9 dimethylation demethylation. This epigenetic modification thereby facilitates C/EBPδ-associated transcriptional activation of adipogenic genes. Conversely, PHF2 deficiency suppresses the expression of adipogenic genes associated with the C/EBPδ-dependent differentiation program [28]. Taken together, protein–protein interactions and epigenetic regulation constitute important additional layers of regulation that facilitate the functional activity of C/EBPδ during adipocyte differentiation.

### 2.4. Endogenous Inhibitory Mechanisms Controlling the Timing of Differentiation Initiation

In parallel with the positive regulatory signals that drive adipocyte differentiation, preadipocytes also maintain endogenous inhibitory mechanisms that help preserve their undifferentiated state. Preadipocyte factor 1 (Pref-1) has been shown to activate the extracellular signal-regulated kinase (ERK) signaling pathway, thereby upregulating the expression of SRY-box transcription factor 9 (Sox9). Sox9 can directly bind to the promoter regions of C/EBPβ and C/EBPδ and repress their transcriptional activity [29,30]. This inhibitory mechanism helps prevent premature initiation of adipocyte differentiation in the absence of appropriate external cues, thereby restraining early induction of adipogenic transcription factors until permissive differentiation signals are present.

Taken together, the upstream regulation of C/EBPδ is not governed by a single signaling input but rather arises from a multilayered regulatory network comprising differentiation-inducing signals, protein homeostasis control, cooperative activation, and endogenous inhibitory mechanisms. This integrated regulatory framework helps coordinate the timely induction and proper functional activity of C/EBPδ during the early phase of adipocyte differentiation, allowing it to respond dynamically to distinct physiological contexts and to contribute to the orderly initiation of downstream transcriptional programs.

## 3. Regulation of Adipocytes by C/EBPδ Under Different Physiological and Pathological Conditions

### 3.1. Overview of Downstream Functions of C/EBPδ in Adipocytes

**Figure 2:**
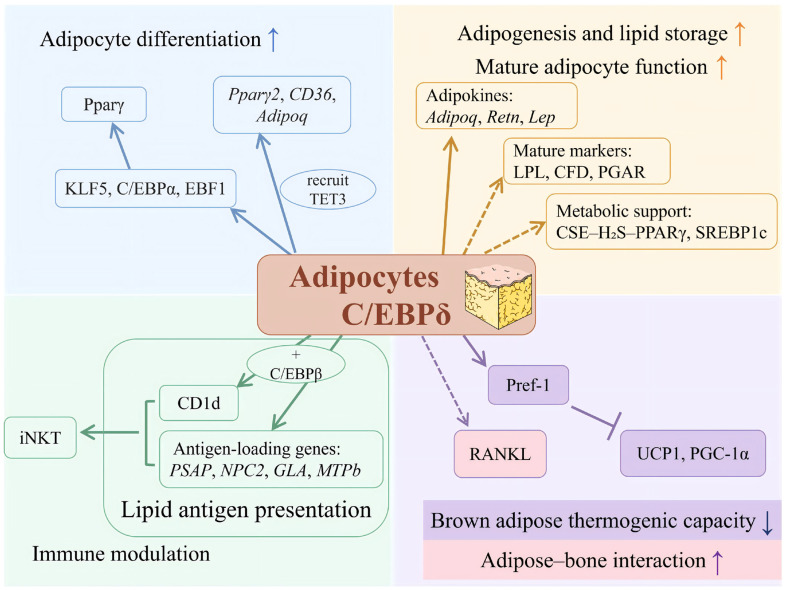
Downstream functional framework associated with C/EBPδ in adipocytes under different physiological and pathological contexts. 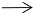 promotion; 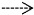 indirect effect; 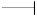 inhibition; Upward arrows indicate increased activity or upregulation, whereas downward arrows indicate decreased activity or downregulation under the indicated conditions. PPARγ: Peroxisome proliferator-activated receptor γ; KLF5: Krüppel-like factor 5; C/EBPα: CCAAT/enhancer-binding protein α; EBF1: Early B-cell factor 1; CD36: Cluster of differentiation 36; TET3: ten-eleven translocation methylcytosine dioxygenase 3; Adipoq: Adiponectin; Retn: Resistin; Lep: Leptin; LPL: Lipoprotein lipase; CFD: Complement factor D; PGAR: ANGPTL4 (angiopoietin-like 4, previously designated PGAR); CSE: Cystathionine γ-lyase; SREBP1c: Sterol regulatory element-binding protein 1c; CD1d: Cluster of differentiation 1d; PSAP: Prosaposin; NPC2: Niemann–Pick disease type C2 protein; GLA: Galactosidase alpha; MTPb: Microsomal triglyceride transfer protein β; iNKT: Invariant natural killer T cell; Pref-1: Preadipocyte factor 1; RANKL: Receptor activator of nuclear factor κB ligand; UCP1: Uncoupling protein 1; PGC-1α: Peroxisome proliferator-activated receptor γ coactivator 1α.

C/EBPδ is an important early inducer of adipocyte differentiation, and its regulatory role spans multiple key stages, including the initiation of differentiation, progression of the transcriptional cascade, and the establishment of mature adipocyte characteristics. Accumulating evidence indicates that C/EBPδ plays an important regulatory role across multiple stages of this process. From the perspective of its downstream functions, the role of C/EBPδ extends beyond promoting adipocyte differentiation; it is also linked to the development of metabolic capacity and the functional maturation of adipocytes. This section provides an overview of the major functional characteristics of C/EBPδ during adipocyte differentiation, thereby establishing the basis for the subsequent discussion of its roles under different functional contexts. Figure 2 summarizes the changes in C/EBPδ expression under various physiological and pathological conditions and their corresponding impacts on adipose tissue-related functions.

During the early stage of adipogenic induction, C/EBPδ expression can be rapidly upregulated within several hours following stimulation with the MDI differentiation cocktail. Notably, a temporal gap exists between the initial increase in C/EBPδ expression and the full execution of its transcriptional regulatory activity. After induction, C/EBPδ participates in early transcriptional regulation; however, the full activation of its transcriptional function and the effective initiation of downstream differentiation programs are typically closely associated with the completion of MCE in preadipocytes. Loss of C/EBPδ impairs MCE and consequently is associated with impaired subsequent adipocyte differentiation [10,31]. This characteristic indicates that the activation and expression of C/EBPδ are tightly coordinated with cell-cycle–related events during early adipogenesis, which also helps explain why C/EBPδ is widely regarded as a key regulator in adipocyte biology.

At the level of transcriptional regulation, C/EBPδ occupies an early upstream position in the adipogenic hierarchy and contributes to the establishment of a core regulatory network that drives adipocyte differentiation through the induction of several key transcription factors. Krüppel-like factor 5 (KLF5) is induced by early C/EBP factors, including C/EBPβ and C/EBPδ, and subsequently cooperates with them to promote activation of PPARγ2, thereby transmitting differentiation signals from the early stage toward terminal maturation [32]. Early B-cell factor 1 (EBF1) has been identified as a downstream target of early C/EBP activity, including the cooperative action of C/EBPβ and C/EBPδ. Its expression further enhances the transcriptional programs associated with C/EBPα and PPARγ, ultimately contributing to the stabilization of the gene expression profile characteristic of mature adipocytes [33]. During this process, C/EBPα is induced downstream of early C/EBP activity and is further reinforced by EBF1, forming a positive transcriptional feedback loop that amplifies adipogenic signaling. Notably, C/EBPδ and C/EBPβ together constitute a core early induction module in adipogenesis. Once they acquire DNA-binding capacity, these factors cooperatively contribute to the activation of C/EBPα and PPARγ2, thereby acting as an important transcriptional node that drives adipocyte differentiation from lineage commitment toward terminal maturation [34].

At the epigenetic level, C/EBPδ cooperates with the DNA demethylase ten-eleven translocation methylcytosine dioxygenase 3 (TET3) to regulate adipogenic gene expression. C/EBPδ can recruit TET3 to C/EBP-binding sites in the promoter regions of major adipogenic genes, such as *Pparγ2*, *Cd36*, and *Adipoq*, thereby promoting DNA demethylation at these loci and enhancing the transcriptional activity of the corresponding target genes. This mechanism appears to be important for maintenance of the adipogenic program. In aging tissues, reduced TET3 levels, despite relatively stable C/EBPδ expression, lead to diminished demethylation capacity at these target gene promoters, which has been associated with the age-related decline in adipogenic potential [35]. These findings indicate that the functional activity of C/EBPδ depends not only on its own induction but also on the coordinated action of epigenetic regulatory factors.

At the level of downstream phenotypes and metabolic regulation, the available evidence suggests that C/EBPδ contributes to the acquisition of lipid storage capacity in mature adipocytes through its participation in early adipogenic transcriptional programs and their downstream metabolic outputs. With respect to downstream effector genes, early C/EBP factors, including C/EBPδ, have been found at the promoters of several adipocyte-associated genes during adipogenic induction, including adiponectin (*Adipoq*), resistin (*Retn*), and leptin (*Lep*), whereas mature adipocyte markers such as LPL, ANGPTL4 (formerly known as PGAR), and CFD are more appropriately viewed as downstream outputs of adipogenic transcriptional programs to which C/EBPδ contributes. Together, these genes contribute to the establishment of the characteristic phenotype and functional properties of mature adipocytes [36,37]. In terms of metabolic support, C/EBPδ appears to be linked to the establishment of metabolic programs associated with adipocyte maturation through multiple downstream pathways. On the one hand, the cystathionine γ-lyase (CSE)/hydrogen sulfide (H_2_S) axis has been shown to support adipogenesis and PPARγ activity, and may intersect with early C/EBP-dependent transcriptional networks. H_2_S subsequently enhances the transcriptional activity of PPARγ through S-sulfhydration, which in turn promotes the expression of adipogenesis-related genes and facilitates lipid accumulation [38]. On the other hand, sterol regulatory element-binding protein 1c (SREBP1c) expression during adipogenesis is regulated by C/EBP transcription factors, with early contributions from C/EBPβ/δ and later predominance of C/EBPα, thereby providing transcriptional support for metabolic programs involved in fatty acid and triglyceride synthesis [39]. Collectively, these metabolism-related genes and signaling axes represent metabolic programs that intersect with C/EBPδ-associated adipogenic regulation, thereby facilitating the orderly progression of adipocyte differentiation and the establishment of the mature adipocyte phenotype.

The function of C/EBPδ also exhibits marked tissue specificity. In fetal and neonatal brown adipose tissue, C/EBPδ can directly bind to and activate the promoter of preadipocyte factor 1 (Pref-1), thereby promoting its transcriptional upregulation. Elevated Pref-1 expression subsequently suppresses the induction of thermogenic genes, including uncoupling protein 1 (UCP1) and peroxisome proliferator-activated receptor gamma coactivator 1-alpha (PGC-1α), ultimately impairing the development of thermogenic capacity in brown adipocytes. Glucocorticoids can further enhance this regulatory effect by inducing C/EBPδ expression and strengthening its transcriptional activation of Pref-1, thereby inhibiting the thermogenic differentiation program of brown adipocytes [40]. The regulatory pattern highlights the functional divergence of C/EBPδ in the development of white and brown adipose tissues. While C/EBPδ is generally associated with promotion of adipogenic differentiation in white adipocyte models, it restricts thermogenic capacity during brown adipose tissue development, suggesting that its functional output is strongly shaped by depot-specific and developmental context. Such differences likely reflect the influence of tissue type and local microenvironment, underscoring the functional diversity of C/EBPδ.

Notably, downstream targets of C/EBPδ also participate in functional interactions between adipocytes and other tissues, a phenomenon that is particularly evident during adipogenesis within the bone marrow microenvironment. Receptor activator of nuclear factor-κB ligand (RANKL) can be induced during adipogenesis through the action of C/EBPβ and/or C/EBPδ. Although RANKL does not directly participate in adipocyte differentiation, its expression enables differentiating adipocytes to influence osteoclastogenesis, thereby establishing an important biological link between adipogenesis and bone remodeling [41]. This finding broadens the understanding of adipocyte function and suggests that C/EBPδ may contribute to functional crosstalk between adipose tissue and the bone microenvironment.

From a systems-level perspective, the current evidence supports the view that C/EBPδ participates in two broad and partially overlapping aspects of adipocyte biology, encompassing differentiation initiation and metabolic support. Its role in differentiation initiation is reflected in its function as an early initiator of adipogenic transcriptional events, thereby influencing whether preadipocytes proceed into the differentiation program. In contrast, its role in metabolic support is manifested through association with metabolic pathways such as the CSE/H_2_S axis and the SREBP1c-related lipogenic program, which provide biochemical support for lipid synthesis and storage in mature adipocytes. These two aspects are temporally sequential yet functionally coordinated, together forming a regulatory framework within which C/EBPδ is associated with both adipocyte differentiation and maturation-related metabolic programs. This perspective highlights that C/EBPδ not only participates in initiation of the differentiation program but also is linked to the establishment of metabolic competence in mature adipocytes, thereby providing a conceptual basis for understanding its context-dependent functions under different physiological and pathological conditions.

### 3.2. Immunoregulatory Role of C/EBPδ in Lipid Antigen Presentation

Recent studies have shown that the regulatory functions of C/EBPδ extend beyond adipocyte differentiation and metabolism to the field of immune regulation, particularly through its association with lipid antigen presentation. In this process, lipid antigens are loaded onto cluster of differentiation 1d (CD1d) molecules and subsequently presented to invariant natural killer T (iNKT) cells, thereby influencing the local immune milieu. This finding suggests that, in addition to their metabolic functions, adipocytes may also participate in local immune regulation through specific molecular pathways, further highlighting the multifaceted role of adipose tissue in systemic homeostasis. Evidence indicates that early adipogenic induction of C/EBPβ and C/EBPδ is accompanied by increased expression of CD1d and related lipid antigen presentation machinery genes. As a non-classical major histocompatibility complex-like molecule, CD1d is primarily responsible for presenting lipid antigens to iNKT cells and thus serves as a key molecular bridge in adipose tissue immune regulation. Further studies have demonstrated that C/EBPβ and C/EBPδ are recruited to a C/EBP-responsive region associated with the *CD1d* locus during adipogenesis, thereby supporting CD1d transcriptional activation [42]. At the same time, several genes essential for lipid antigen loading, including prosaposin (*Psap*), Niemann–Pick type C2 (*Npc2*), galactosidase alpha (*Gla*), and microsomal triglyceride transfer protein β (*MTPb*), have been identified as components of a lipid antigen presentation program transcriptionally regulated by C/EBPβ and C/EBPδ. These genes are responsible for lipid antigen processing, intracellular trafficking, and loading onto CD1d molecules, collectively providing the molecular basis for an intact lipid antigen presentation pathway. Taken together, this C/EBPβ/δ-associated lipid antigen presentation axis provides a more concrete molecular framework for understanding adipocyte–immune system interactions and suggests that early C/EBP activity, including C/EBPδ, may participate in the development of obesity-associated metaflammation. This, in turn, offers a new perspective and potential molecular targets for investigating the link between adipose tissue dysfunction and metabolic disease.

### 3.3. Regulation of Adipocytes by C/EBPδ Under Inflammatory and Stress-Related Conditions

Under inflammatory or stress-related microenvironmental changes—such as exposure to proinflammatory cytokines, hypoxia, or oxidative stress—the expression of C/EBPδ in adipose tissue is often altered and may be associated with changes in either the differentiation capacity of adipocyte progenitors or the lipid storage programs of adipocyte-related cells. In these contexts, the expression and function of C/EBPδ are often highly dependent on the cellular microenvironment. In many experimental models, persistent or excessive inflammatory and stress signals are associated with disruption of the adipogenic program or suppression of lipid metabolism–related processes. These findings underscore the function of C/EBPδ as a transcriptional regulator that responds to stress-responsive regulatory pathways.

Under inflammatory stimulation, the adipogenic capacity of preadipocytes is often impaired, indicating that inflammatory signaling can substantially interfere with the adipogenic program. Studies have shown that treatment of preadipocytes with tumor necrosis factor-α significantly reduces C/EBPδ expression and concomitantly suppresses the expression of PPARγ2, thereby inhibiting the early differentiation process [43]. These findings suggest that, under inflammatory conditions, tumor necrosis factor-α may disrupt the C/EBPδ–PPARγ2 transcriptional regulatory axis, ultimately impairing the normal initiation of adipocyte differentiation.

Chemokine-related signaling also regulates C/EBPδ in a context-dependent manner. Monocyte chemoattractant protein-1 (MCP-1) can induce the early upregulation of its downstream effector, MCP-1-induced protein 1. In 3T3-L1 cells, MCP-1-induced protein 1 overexpression has been associated with increased expression of C/EBPβ and C/EBPδ during adipogenic differentiation [44]. In addition, the signaling axis involving C-X-C motif chemokine ligand 3 and its receptor C-X-C chemokine receptor type 2 has been reported to promote adipocyte differentiation in models such as 3T3-L1 cells, in association with increased expression of both C/EBPβ and C/EBPδ. Conversely, knockdown or inhibition of C-X-C motif chemokine ligand 3 or C-X-C chemokine receptor type 2 attenuates the differentiation process [45]. These findings indicate that different chemokine signals may be associated with the upregulation of C/EBPδ; however, their biological significance should be interpreted in the context of the specific upstream stimuli and the broader regulatory network.

Various stress conditions can also influence adipocyte differentiation or adipogenesis by altering the expression of C/EBPδ. In bone marrow–derived mesenchymal stem cells, extreme hypoxia (0.2% O_2_) has been reported to upregulate C/EBPδ expression, accompanied by increased lipid droplet accumulation and enhanced expression of multiple adipogenesis-related genes, suggesting that severe hypoxic conditions can promote adipogenic differentiation in this mesenchymal progenitor model [36]. Similarly, oxidative stress, such as treatment with hydrogen peroxide, has been associated with reciprocal alterations in C/EBPα and C/EBPδ isoforms together with impaired GLUT4-related transcriptional regulation in 3T3-L1 adipocytes [46]. Collectively, these findings suggest that the elevation of C/EBPδ under stress conditions may primarily reflect stress-responsive transcriptional remodeling, and current evidence does not support a uniform causal interpretation linking increased C/EBPδ to a common adipogenic outcome across different stress settings.

C/EBPδ also occupies a key regulatory position in paracrine and extracellular matrix–associated signaling within the tissue microenvironment. Studies have shown that connective tissue growth factor, in a TGF-β-dependent manner, as well as exogenous TGF-β1, inhibits the early upregulation and nuclear localization of C/EBPβ and C/EBPδ, accompanied by reduced expression of adipogenic genes and inhibition of adipocyte differentiation [47]. In contrast, in rat tendon stem cells, prostaglandin E_2_ can upregulate insulin-like growth factor-1 expression through a cAMP/PKA/C/EBPδ-dependent pathway. Insulin-like growth factor-1, acting in cooperation with bone morphogenetic protein-2, subsequently promotes adipogenic differentiation in this tendon-derived stem cell model [48]. These findings further indicate that C/EBPδ does not function as a single determinant of adipogenic fate but rather acts as an important responsive node within a broader microenvironmental signaling network.

### 3.4. Regulation of Adipocytes by C/EBPδ Under Changes in Metabolic and Nutritional Status

The metabolic and nutritional status of adipocyte progenitors and adipose tissue can markedly influence the expression of C/EBPδ and the functional readouts associated with its activity. Unlike the rapid regulatory events that occur during the early phase of adipocyte differentiation, regulation of C/EBPδ under metabolic and nutritional conditions more often reflects adaptive cellular responses to long-term or systemic energy states. In such contexts, the functional consequences of C/EBPδ activity are not necessarily limited to promoting differentiation; rather, they are more closely associated with changes in adipogenic capacity and the functional properties of adipose cells under distinct metabolic states. This characteristic further highlights C/EBPδ as a transcription factor that is highly responsive to metabolic cues.

Under conditions of systemic energy restriction, C/EBPδ expression can be upregulated, whereas adipogenesis- and lipogenesis-related programs are often concurrently suppressed, consistent with a transcriptional state less compatible with active adipogenesis and lipid storage. Studies have shown that systemic fasting elevates C/EBPδ expression in adipose tissue while reducing the expression of some adipogenic regulators, including PPARγ2, suggesting a fasting-associated shift in adipogenic transcriptional state [49]. These findings suggest that elevated C/EBPδ expression under energy-deficient conditions is more consistent with a fasting-adaptive transcriptional state than with an active adipogenic program.

Various metabolically active small molecules can also regulate C/EBPδ expression and influence adipocyte differentiation outcomes by altering the cellular metabolic context. Treatment of undifferentiated mesenchymal stem cells with all-trans retinoic acid suppresses C/EBPδ expression and is accompanied by downregulation of key adipogenic regulators such as PPARγ, thereby inhibiting adipogenic differentiation in this progenitor model [50]. Similarly, isoniazid, a drug capable of altering cellular metabolic status, reduces C/EBPδ expression and inhibits the induction of adipogenesis-related genes, accompanied by impaired adipocyte differentiation [51]. Collectively, these findings indicate that metabolic interventions often affect the final differentiation and maturation of adipocytes in parallel with changes in C/EBPδ expression.

Notably, aging-associated metabolic changes can also alter the regulatory characteristics of C/EBPδ. Studies have shown that the expression of C/EBPδ during the early phase of adipocyte differentiation decreases with advancing age and is often accompanied by reduced differentiation capacity of adipocyte progenitors, primarily manifested as impaired adipogenesis [52]. These observations indicate that, under conditions of long-term metabolic and physiological alterations, the functional implications of changes in C/EBPδ expression are highly context-dependent. Therefore, the biological significance of C/EBPδ expression dynamics should be interpreted within the specific physiological or pathological context.

### 3.5. Regulation of Adipocytes by C/EBPδ Under Physiological Stimuli Such as Exercise and Mechanical Loading

Physiological activity at the whole-body level, particularly stimuli related to exercise and mechanical loading, can also influence the expression profile of C/EBPδ in adipose tissue and be accompanied by changes in adipocyte differentiation and adipogenesis-related programs. This phenomenon suggests that the role of C/EBPδ is not confined to local intracellular regulation, but may also be involved in systemic physiological adaptation. Studies have shown that acute exercise can markedly alter C/EBPδ expression in adipose tissue. In acute exercise models, C/EBPδ expression is upregulated after exercise, whereas the expression of adipogenesis-related genes is suppressed, indicating an exercise-responsive transcriptional adaptation in adipose tissue rather than direct evidence that C/EBPδ itself mediates the reduction in adipogenic capacity [53]. These findings suggest that, under conditions of increased short-term energy expenditure, upregulation of C/EBPδ is more consistent with an acute exercise-responsive transcriptional adaptation in adipose tissue.

However, under exercise models involving mechanical loading, the expression pattern of C/EBPδ appears to differ. Studies have shown that high-impact jumping training significantly reduces bone marrow adipose tissue volume, while no significant changes were detected in the protein or mRNA expression of key adipogenic transcription factors, including PPARγ2 and the C/EBP family proteins C/EBPα, C/EBPβ, and C/EBPδ [54]. These findings suggest that the reduction in bone marrow adiposity induced by mechanical loading is not necessarily mediated through altered expression of classical adipogenic transcription factors such as C/EBPδ. Related studies further indicate that the decrease in bone marrow fat following high-impact jumping training is more likely attributable to the upregulation of osteogenesis-related factors, which may secondarily suppress bone marrow adipogenesis.

Taken together, different types of physiological stimuli can influence adipocyte fate through distinct mechanisms, and C/EBPδ does not occupy an equally central role in all contexts. Acute exercise–induced short-term negative energy balance may be accompanied by changes in C/EBPδ-associated transcriptional responses and suppression of adipogenic gene expression. In contrast, the reduction in adiposity induced by mechanical loading may partially bypass the C/EBPδ node and instead be mediated through mechanical signaling and alterations in the local microenvironment. These differences further suggest that the function of C/EBPδ should be interpreted in light of the specific type of stimulus and the relevant tissue context.

## 4. Feasibility and Challenges of Targeting C/EBPδ for the Intervention of Adipocyte-Related Diseases

An important issue emerging from current studies is that many reported interventions that “affect C/EBPδ expression” do not act directly on C/EBPδ itself. Instead, they more often alter upstream signaling pathways, metabolic states, or the local microenvironment, thereby indirectly leading to changes in C/EBPδ expression. In other words, based on the evidence currently available, C/EBPδ appears to function more as a transcriptional response node triggered by external stimuli than as a validated drug target amenable to direct and specific pharmacological intervention. For example, isorhamnetin inhibits adipocyte differentiation primarily through downregulation of PPARγ and C/EBPα, without significantly affecting C/EBPβ or C/EBPδ [55], indicating that adipocyte differentiation can be suppressed even in the absence of a clear change in C/EBPδ expression. On the other hand, some studies have shown that when certain polyphenol mixtures, such as Oligonol, inhibit adipogenesis through signaling pathways including Akt–mTOR, multiple adipogenesis-related transcription factors exhibit an overall downward trend [56], with changes in C/EBPδ representing only one component of a broader network response. Moreover, the direction of C/EBPδ regulation and the resulting differentiation outcome are not always consistent across different interventions. Some treatments inhibit adipocyte differentiation without significantly altering C/EBPδ expression, whereas others suppress adipogenesis in parallel with C/EBPδ downregulation. These observations further indicate that the interpretive value of intervention studies depends not only on whether C/EBPδ expression changes, but also on whether C/EBPδ is directly perturbed, indirectly affected as part of a broader network response, or functionally required for the observed phenotype. Representative intervention factors reported to date are summarized in Table 1. Notably, most of these studies are informative at the level of pathway context or expression association, whereas adipocyte-specific evidence directly demonstrating C/EBPδ as the required mediator of the observed phenotype remains limited.

Although small-molecule strategies that directly target C/EBPδ remain underdeveloped in the field of adipose biology, experimental evidence has demonstrated that directly altering C/EBPδ levels at critical stages of adipocyte differentiation can substantially influence early adipogenic progression. Hishida et al. showed that RNA interference (RNAi)-mediated knockdown of C/EBPδ in 3T3-L1 preadipocytes significantly suppressed early proliferative events and the subsequent differentiation process during adipogenesis [10], indicating that C/EBPδ is functionally required during the early phase of adipogenic progression. Such genetic evidence suggests that C/EBPδ itself is experimentally actionable, although this does not yet establish direct pharmacological tractability in adipocytes.

Prototype strategies that directly interfere with C/EBPδ function have already emerged in other disease research contexts. In tumor studies, researchers have developed dominant-negative peptides conjugated with a cell-penetrating peptide, which target the leucine zipper domain of transcription factors such as C/EBPδ. By disrupting homo- or heterodimerization and the subsequent DNA-binding activity, these peptides suppress the transcriptional regulatory function of C/EBPδ [69]. This strategy has shown preclinical therapeutic potential in several tumor cell models and animal studies, although the available evidence remains largely confined to the preclinical stage [70]. While these findings cannot be directly translated to the adipocyte system, they provide a conceptual precedent for direct interference with C/EBPδ function rather than adipocyte-specific validation.

In addition to directly blocking its activity, modulation of the upstream pathways and expression homeostasis of C/EBPδ may represent another conceptually feasible therapeutic strategy. Previous studies in systems such as macrophages have shown that C/EBPδ expression and function can be regulated at multiple levels, including transcriptional control by upstream transcription factors, post-transcriptional regulation of mRNA stability, and post-translational regulation of protein degradation [71,72]. Although these findings cannot substitute for direct validation in adipocytes, they may nevertheless provide a conceptual framework for achieving temporally and cell type-selective regulation of C/EBPδ in adipocyte-related systems.

Despite the clear theoretical rationale and the availability of insights from other biological systems, the translation of C/EBPδ into a clinically actionable and precise therapeutic target still faces multiple challenges. First, transcription factors have long been regarded as a class of targets with limited druggability. C/EBPδ is a member of the basic leucine zipper transcription factor family, and its activity relies on the formation of homo- or heterodimers. However, the relevant protein–protein interaction interfaces are typically relatively flat and lack the well-defined binding pockets that are readily targeted by conventional small molecules, making high-affinity and highly selective direct intervention difficult to achieve [72]. Second, C/EBPδ exhibits pronounced cell type specificity, tissue specificity, and context dependence. Previous studies have suggested that, in addition to its roles in adipocyte differentiation and adipogenesis, C/EBPδ also participates in a wide array of biological processes, including inflammatory and immune responses, oxidative stress, lineage specification, and cell proliferation [16]. In the cardiovascular system, aberrant C/EBPδ expression has also been implicated in diseases such as myocardial infarction and atherosclerosis, although its precise roles across different tissues and disease stages remain to be fully elucidated [72,73,74]. Therefore, in the absence of tissue- or cell type-specific delivery and regulatory strategies, systemic targeting of C/EBPδ may lead to unintended off-target effects and safety concerns.

Overall, C/EBPδ holds clear theoretical value as an intervention target, but it remains at a stage characterized by substantial mechanistic understanding, preliminary tool development, and limited translational progress. Future studies should not only further define the temporal and context-dependent roles of C/EBPδ in adipocytes, but also focus on developing more cell type-specific and context-oriented intervention strategies, thereby improving its translational potential in obesity and other adipocyte-related disorders.

## 5. Conclusions and Perspectives

Current evidence indicates that C/EBPδ is an important transcriptional regulator within the regulatory network of adipose tissue biology. As a rapidly responsive factor induced during the early phase of adipocyte differentiation, C/EBPδ occupies an early regulatory position within adipogenic transcriptional programs and links upstream differentiation-related cues with downstream core adipogenic regulators, including PPARγ and C/EBPα. One of the most notable regulatory features of C/EBPδ is its strong context dependence and tissue heterogeneity. In other words, the function of C/EBPδ is not manifested as a single, linear pro-differentiation effect; rather, it is highly dependent on the surrounding signaling environment, cellular state, and tissue microenvironment. This feature also represents the central theme underlying the biological significance of C/EBPδ discussed throughout this review.

In recent years, the significance of C/EBPδ has extended beyond its role in adipocyte differentiation alone and increasingly encompasses immune regulation in adipose tissue, maintenance of metabolic homeostasis, and the study of related disease mechanisms. Nevertheless, the current body of evidence remains insufficient to define C/EBPδ as a direct therapeutic target for adipose-related diseases. In most physiological and pathological contexts, C/EBPδ appears to function more as a transcriptional response node that enables adipocytes to adapt to environmental changes, rather than as a single decisive driver, particularly in light of the still limited adipocyte-specific direct validation currently available. At the same time, the intrinsic druggability limitations of transcription factors continue to pose substantial barriers to its clinical translation.

In view of these considerations, future research may focus on two main directions. First, systematic clarification of C/EBPδ-specific, shared C/EBP-family, and context-dependent regulatory mechanisms in adipocytes is needed to further define its functional roles and to identify more stable and druggable key nodes within these networks. Second, based on such mechanistic insights, efforts should be directed toward developing adipocyte-selective tools for the precise regulation of C/EBPδ. Overall, the value of studying C/EBPδ lies in providing an important entry point for understanding how adipocytes respond to internal and external environmental changes, and in offering new theoretical insights for elucidating the mechanisms of adipose-related diseases and for exploring precision intervention strategies.

## Figures and Tables

**Table 1:** Representative exogenous interventions associated with altered C/EBPδ expression and adipogenic outcomes.

Type of Intervention	Interventions	C/EBPδ Response	Effect on Adipogenic Outcome	References
Drug and metabolism-related small molecules	Carbacyclin	Indirect promotion (pathway-linked)	Promotion of adipocyte differentiation	[57]
	Isoniazid	Indirect inhibition (correlative)	Inhibition of adipocyte differentiation	[51]
	Pioglitazone	Indirect promotion (pathway-linked)	Promotion of adipocyte differentiation	[58]
	All-trans retinoic acid	Indirect inhibition (correlative)	Inhibition of adipocyte differentiation	[50]
	Propranolol	Indirect promotion (correlative)	Promotion of adipocyte differentiation	[59]
	P6 (Pyridone 6; pan-JAK inhibitor)	Indirect inhibition (pathway-linked)	Inhibition of adipocyte differentiation	[19]
	Cholecalciferol	Indirect inhibition (correlative)	Inhibition of early adipogenesis	[60]
	extended-release niacin	Indirect promotion (correlative)	Promotion of adipocyte differentiation	[61]
	Compound C (dorsomorphin; AMPK inhibitor)	Indirect inhibition (pathway-linked)	Inhibition of adipocyte differentiation	[31]
Natural bioactive components	Bouchardatine	Indirect inhibition (correlative)	Inhibition of adipogenesis and early adipocyte differentiation	[62]
	Orientin	Indirect inhibition (pathway-linked)	Inhibition of adipogenesis and adipocyte differentiation	[63]
	Artemisinic acid	Indirect inhibition (correlative)	Inhibition of adipocyte differentiation	[64]
	Monascin	Indirect inhibition (correlative)	Inhibition of adipocyte differentiation	[65]
	Ankaflavin	Indirect inhibition (correlative)	Inhibition of adipocyte differentiation	[65]
Natural mixtures	Xanthigen	Indirect inhibition (pathway-linked)	Inhibition of adipocyte differentiation	[66]
	*Aster spathulifolius*	Indirect inhibition (correlative)	Inhibition of adipocyte differentiation	[67]
	Hot water extract of edible *Chrysanthemum morifolium*	Indirect promotion (correlative)	Promotion of adipocyte differentiation	[68]

## Data Availability

Data sharing is not applicable. No new data were created in this study.

## Abbreviations

The following abbreviations are used in this manuscript:

C/EBPδ	CCAAT/enhancer-binding protein δ
KLF5	Krüppel-like factor 5
EBF1	early B-cell factor 1
C/EBPα	CCAAT/enhancer-binding protein α
PPARγ	peroxisome proliferator-activated receptor γ
CSE/H_2_S	cystathionine γ-lyase/hydrogen sulfide
SREBP1c	sterol regulatory element-binding protein 1c
C/EBPβ	CCAAT/enhancer-binding protein β
IBMX	3-isobutyl-1-methylxanthine
DEX	dexamethasone
MDI	IBMX, DEX and insulin
MCE	mitotic clonal expansion
ATF2	Activating transcription factor 2
ATF4	Activating transcription factor 4
AMPK	AMP-activated protein kinase
cAMP	cyclic adenosine monophosphate
CTCF	CCCTC-binding factor
DDX5	DEAD-box helicase 5
ERK	extracellular signal-regulated kinase
GR	glucocorticoid receptor
GRE	glucocorticoid response element
JNK2	c-Jun N-terminal kinase 2
KCNK10	potassium two-pore domain channel subfamily K member 10
MDM2	murine double minute 2
PHF2	plant homeodomain finger protein 2
PKD1	protein kinase D1
Pref-1	preadipocyte factor 1
Sox9	SRY-box transcription factor 9
14-3-3ζ	14-3-3 zeta protein
TET3	ten-eleven translocation methylcytosine dioxygenase 3
Adipoq	Adiponectin
Retn	Resistin
Lep	Leptin
LPL	Lipoprotein lipase
CFD	Complement factor D
PGAR	ANGPTL4 (angiopoietin-like 4, previously designated PGAR)
CD1d	Cluster of differentiation 1d
PSAP	Prosaposin
NPC2	Niemann–Pick disease type C2 protein
GLA	Galactosidase alpha
MTPb	Microsomal triglyceride transfer protein β
iNKT	Invariant natural killer T cell
RANKL	Receptor activator of nuclear factor κB ligand
UCP1	Uncoupling protein 1
PGC-1α	Peroxisome proliferator-activated receptor γ coactivator 1α
MCP-1	Monocyte chemoattractant protein-1
